# Developing the Ternary ZnO Doped MoS_2_ Nanostructures Grafted on CNT and Reduced Graphene Oxide (RGO) for Photocatalytic Degradation of Aniline

**DOI:** 10.1038/s41598-020-61367-7

**Published:** 2020-03-10

**Authors:** Parisa Ghasemipour, Moslem Fattahi, Behnam Rasekh, Fatemeh Yazdian

**Affiliations:** 1 Chemical Engineering Department, Abadan Faculty of Petroleum Engineering, Petroleum University of Technology, Abadan, Iran; 2 Microbiology and Biotechnology Research Group, Research Institute of Petroleum Industry, National Iranian Oil Company, Tehran, Iran; 3 Department of Life Science Engineering, Faculty of New Science and Technologies, University of Tehran, Tehran, Iran

**Keywords:** Engineering, Materials science, Nanoscience and technology

## Abstract

Transition metal sulfide semiconductors have achieved significant attention in the field of photocatalysis and degradation of pollutants. MoS_2_ with a two dimensional (2D) layered structure, a narrow bandgap and the ability of getting excited while being exposed to visible light, has demonstrated great potential in visible-light-driven photocatalysts. However, it possesses fast-paced recombination of charges. In this study, the coupled MoS_2_ nanosheets were synthesized with ZnO nanorods to develop the heterojunctions photocatalyst in order to obtain superior photoactivity. The charge transfer in this composite is not adequate to achieve desirable activity. Therefore, heterojunction was modified by reduced graphene oxide (RGO) nanosheets and carbon nanotubes (CNTs) to develop the RGO/ZnO/MoS_2_ and CNTs/ZnO/MoS_2_ ternary nanocomposites. The structure, morphology, composition, optical and photocatalytic properties of the as-fabricated samples were characterized through X-ray diffraction (XRD), Fourier Transform Infrared (FTIR), Field Emission Scanning Electron Microscopy (FESEM), Transmission Electron Microscopy (TEM), Energy-Dispersive X-ray (EDX), elemental mapping, Photoluminescence (PL), Ultraviolet–Visible spectroscopy (UV-VIS), and Brunauer-Emmett-Teller (BET) techniques. The photo-catalytic performance of all samples was evaluated through photodegradation of aniline in aqueous solution. The combination of RGO or CNTs into the ZnO/MoS_2_ greatly promoted the catalytic activity. However, the resulting RGO/ZnO/MoS_2_ ternary nanocomposites showed appreciably increased catalytic performance, faster than that of CNTs/ZnO/MoS_2_. Charge carrier transfer studies, the BET surface area analysis, and the optical studies confirmed this superiority. The role of operational variables namely, solution pH, catalyst dosage amount, and initial concentration of aniline was then investigated for obtaining maximum degradation. Complete degradation was observed, in the case of pH = 4, catalyst dosage of 0.7 g/L and aniline concentration of 80 ppm, and light intensity of 100 W. According to the results of trapping experiments, hydroxyl radical was found to be the main active species in the photocatalytic reaction. Meanwhile, a plausible mechanism was proposed for describing the degradation of aniline upon ternary composite. Moreover, the catalyst showed excellent reusability and stability after five consecutive cycles due to the synergistic effect between its components. Total-Organic-Carbon concentration (TOC) results suggested that complete mineralization of aniline occurred after 210 min of irradiation. Finally, a real petrochemical wastewater sample was evaluated for testing the catalytic ability of the as-fabricated composites in real case studies and it was observed that the process successfully quenched 100% and 93% of Chemical Oxygen Demand (COD) and TOC in the wastewater, respectively.

## Introduction

Water scarcity and increasing amount of discharges of effluents from industries as well as agricultural runoff flowing into water resources, all as a consequence of civilization; make the recycle and treatment of water more and more vital. Aniline is a crucial raw and intermediate material that has various applications in the manufacture of polymer herbicides, pharmaceuticals, polyurethane, and dyes. Besides, it is widely produced in petrochemical plants. However, it is a toxic and cancer-causing substance, and its presence in the wastewater of these industries could result in accumulation in trace concentration in water bodies, creating a risk for the public health and wildlife. Therefore, it is urgent to develop a new practical method to minimize and eliminate this pollutant before being released into the environment. Up to now, many methods have been used for treatment of waste water, including membrane filtration, adsorption, ozonation, biological treatment, and electrolysis^1–6^. Efforts by researchers for finding the best methods or approaches to tackle water issues and find suitable alternative for conventional wastewater treatment have led to the discovery and development of the photocatalytic process^7–9^. In the photocatalytic systems, semiconductor-based materials including metal sulfides (ZnS, CdS), metal oxides (Fe_2_O_3_,WO_3_, TiO_2_), etc. are exploited under the illumination of light, and when a photon with sufficient energy reaches the semiconductor surface, it would be excited and consequently the electron-hole pairs would be generated. Then, these photo-induced charges are transferred to the photocatalyst surface where they undergo reduction and oxidation reactions with O_2_ and OH^−^ to produce superoxide anions (.O_2_^−^) and hydroxyl radicals (.OH), which are capable of degrading various hazardous organic compounds^10–15^.

Recently, following the continuous progress in the photocatalysis field, there has been a notable focus on the development of the ideal semiconductor photocatalyst. For his purpose, finding a photocatalyst with a suitable bandgap for absorbing light in the visible region as well as being economically and practically favorable for large-scale deployment was of high request. Previous studies showed that, solar light is an intrinsic inexhaustible supply of environmental energy. It is noteworthy that only 4% of solar light is placed in the UV region, while nearly 46% of its spectrum is placed in the visible region which is far abundant. Hence, developing a robust solar-responsive photocatalyst is urgently required for the next-generation of photocatalysts^16–23^.

So far, transition metal sulfide semiconductors such as CdS, MoS_2_, and WS_2_ have achieved much attention because of their narrow bandgap and their ability to separate charges under the visible light^24–28^. MoS_2_ with a 2D layered structure and a narrow bandgap of 2 eV is capable to absorb noticeable amount of light, with excellent thermal and chemical stability. This semiconductor has demonstrated a great potential towards water splitting and degradation of pollutants. Nevertheless, pure MoS_2_ has a major demerit, which is poor quantum yield caused by the insufficient lifetime of photo-induced pairs of electron-hole, which is not considered to be desirable for the redox reaction. It also has a strong tendency towards aggregation and restacking which in turn leads to a significant decrease in the accessible surface area^29–32^.

ZnO, as one of the most studied semiconductor photocatalysts, has been extensively applied in degradation of pollutants because it is a metal-free, nontoxic, and low-cost photocatalyst and is effective for almost full mineralization of pollutants. ZnO with a bandgap of 3.37 eV has limited application in the presence of visible light. Furthermore, rapid recombination of photo-induced pairs of electron-hole in the photocatalytic reaction would cause a reduction in its efficiency. The previous studies showed that coupling this semiconductor with a narrow bandgap semiconductor has resulted in an improvement in its light absorption range and yielded better catalytic results^33–40^. For instance, Tan *et al*. reported that incorporation of ZnO into MoS_2_ could result in easy transfer of electrons to the ZnO surface. As a result, the charges could be separated effectively, and finally, the photocatalytic performance would be improved^41^.

A number of studies have been conducted on graphene, as a 2D carbon sheet; for the fabrication of graphene-based semiconductors due to its exceptional features and also graphene (GR) is considered to be one of the main materials to be used in environmental remediation technologies in future, particularly for wastewater treatment. The superior conductivity property of graphene causes the facilitation regarding the transfer of produced electrons from photocatalyst surface to its conjugated plane. Graphene is known to be a zero bandgap material with low Fermi level, therefore; it acts as an electron storage which receives electrons from the conduction band of the photocatalyst which in turn limits the recombination of electrons and holes. As a consequence, the photocatalytic performance could be boosted^15,42–46^. Yu *et al*. in their study showed that incorporation of graphene into MoS_2_ nanosheets caused a significant effect on the activity of MoS_2_, and the GR/MoS_2_ hetero-structures showed more than twice photocatalytic performance for H_2_ generation than bare MoS_2_ nanosheets^47^. Yuan *et al*. in a study improved the electrical conductivity and photocatalytic activity of MoS_2_ by coupling it with reduced graphene nanosheets. The RGO sheets acted as a charge transfer highway caused the easy transfer of charges resulting in the limitation in the recombination of electron and holes and improvement of H_2_ evolution^48^. Interestingly, many researchers have emphasized the extraordinary effect of graphene on improving the photocatalytic performance of semiconductor which is somehow similar to that of CNT-based photocatalysts. However, the effect of these carbon-based materials on semiconductor photocatalysts such as MoS_2_ has been investigated separately, and usually a thoughtful comparison between graphene and CNT-semiconductor photocatalysts and their ability for degradation of organic pollutants has been left untouched^49–52^.

In the present study, we have focused on developing novel ternary nanocomposites by combining RGO or CNTs with ZnO to enhance the photocatalytic activity of MoS_2_ for degradation of aniline under exposure to visible light. To the best of our knowledge, there have been no studies which used these nanocomposites for photocatalytic degradation of organic pollutants, especially aniline. Besides, no detailed studies can be found which systematically compare the impact of RGO and CNTs in composite with ZnO/MoS_2_. In addition, the effects of various parameters including the concentration of aniline, the pH of the aniline solution, the catalyst dosage amount, and the presence of radical scavengers were also evaluated. Furthermore, the reaction was repeated several times in order to determine the reusability of the composites. Moreover, a probable mechanism was proposed for describing the process of photocatalysis. Finally, the efficiency of the fabricated samples was evaluated in terms of wastewater treatment using a real wastewater sample.

## Materials and experimental procedure

### Materials

Sodium molybdate dihydrate (Na_2_MoO_4_.2H_2_O) and thiourea (CH_4_N_2_S) as precursors of MoS_2_ and zinc acetate as a precursor of ZnO were purchased from Sigma-Aldrich, USA. Aniline was supplied from Merck, Germany. Graphene oxide (GO) and Multi-walled carbon nanotubes (MWCNTs, with high purity >97%, OD < 7 nm, ID = 2–5 nm, length = 10–30 µm) were obtained from US Research Nanomaterials Inc., USA. Other chemicals such as sodium hydroxide (NaOH), hydrochloric acid (HCl), ethanol, p-benzoquinone, isopropanol, and disodium ethylene diamine tetra acetic acid were purchased from Merck, Germany.

### Wastewater sampling method

A real petrochemical wastewater sample was prepared from the effluent of a petrochemical company, in Iran. The sample was kept in a 10 L container at 4 °C^53^.

### Synthesis of MoS_2_ nanosheets

For preparation of MoS_2_ nanosheets, 1.9 g of thiourea, as a source of sulfur content was dissolved in 80 mL of DI water. Simultaneously, 1.03 g of sodium molybdate was added to this solution accompanied by constant stirring. Next, the mixture was poured into a 100 mL Teflon-sealed autoclave and was heated for 15 hour at 200 °C. The black product was filtered and was washed several times using ethanol and DI water. Finally, the sample was annealed for 2 hour at 700 °C to improve its crystallinity^34^.

### Synthesis of ZnO nanorods

In order to develop ZnO nanorods, 2.195 g of Zn(CH_3_COOH)_2_ was dissolved in 80 mL of ethanol. Then, 0.8 g of NaOH was added to the solution and was stirred for 20 min. Next, the solution was poured into a 100 mL Teflon-sealed autoclave and was heated at 150 °C for 12 hour. After the reaction, ZnO nanorods were collected through centrifugation and were washed using ethanol and water. In order to improve the crystallinity and to obtain the Wurtzite phase, the sample was annealed at 700 °C for 2 hour^53,54^.

### Synthesis of binary and ternary samples

CNT/ZnO/MoS_2_ nanocomposite was synthesized by a typical hydrothermal method. First, a certain amount of CNTs (0.07, 0.1 and 0.13 gr CNTs) was dispersed into a mixture of ethanol (40 mL) and water (40 mL) through ultrasonic treatment at 60 watts for 3 hour. Then, 0.8 g of MoS_2_ and 0.2 g of ZnO were added to the above-mentioned solution and were sonicated again for 1 hour. The resulting solution was put into a 100 mL hydrothermal autoclave and was kept at 250 °C for 15 h. Then, the prepared composite was centrifuged and was washed several times using distilled water and was dried at 60 °C overnight. Finally, the nanocomposite was annealed for 2 hour at 300 °C for obtaining intimate contact between the components. Similar procedure was carried out to prepare samples of RGO/ZnO/MoS_2_ nanocomposite. Also, the binary nanocomposite (ZnO/MoS_2_) was prepared without using carbonaceous materials^55^.

### Characterization

X-ray diffraction (XRD) experiment was conducted using PHILIPS PW1730 (Netherland) Diffractometer with Cu K_*α*_ radiation in scanning range of 2*θ* = 5°–70°. A THERMO AVATAR (USA) Fourier Transform Infrared (FTIR) spectrometer was utilized to obtain the spectra with the wavelength range of 400–4000 cm^−1^. The surface morphology of the samples was evaluated using Field Emission Scanning Electron Microscopy (FESEM-FEI MOVA NANOSEM 450, USA). The Transmission Electron Microscopy (TEM) was obtained by Zeiss EM 900 apparatus. Energy-Dispersive X-ray (EDX) spectroscope and elemental mapping were applied in order to determine the composition of the samples. The Barrett-Joyner-Halenda (BJH) method was applied to determine the pore size and Brunauer-Emmett-Teller (BET) method was employed to assess the specific surface area based on the nitrogen adsorption/desorption isotherms using BELSORP MINI II (Japan) instrument. All photocatalysts samples were degassed at 120 °C for 2 hour before the analysis. Photoluminescence (PL) spectra were obtained using AvaSpec-2048 apparatus. UV-vis spectra of the samples were analyzed by an Avantes (AvaSpec-2048-TEC) spectrophotometer in the range of 200–1200 nm. The mineralization was studied through determination of Total-Organic-Carbon-concentration (TOC) using TOC-VCSH Shimadzu analyzer, Japan. The components of real wastewater sample prepared from the effluent of the petrochemical company were identified using Agilent 7890 GC-MS, USA. COD determination was done using open-reflux method, based on the available standard methods for water and wastewater examination.

### Experimental procedure

Aniline was applied as a study model to measure the photocatalytic performance of samples at ambient temperature. First, all the as-fabricated samples were evaluated under the same condition in order to select the best photocatalyst in terms of photocatalytic performance. Initially, 1.2 g of the nanocomposite was added into 100 mL of 100 ppm aniline solution at pH = 7. Prior to the reaction, the solution was vigorously stirred in dark to establish the adsorption-desorption equilibrium time between the photocatalyst and aniline solution. Then, the experiment was initiated under the exposure to light for 120 min using two 50 W LED lamps, as a source of visible light and they were placed 10 cm away from the reaction media. During the reaction, 2 mL of aniline solution was taken at a regular interval of 15 min using a syringe and after centrifugation, the variation in the aniline concentration was monitored using UV-vis spectrophotometer (Unico, USA) at maximum absorbance of 230 nm (λ_max_ = 230 nm). The results showed that RGO10%/ZnO/MoS_2_ had the highest photocatalytic performance among all samples, therefore; it was chosen for performing the optimization of the operational parameters. Finally, the effect of operational parameters such as pH, the catalyst dosage amount, and aniline concentration was evaluated during the course of study. The value for removal and total mineralization of aniline in the photocatalytic degradation process was calculated by the following equations^56^:

1 $${\rm{Aniline}}\,{\rm{removal}}=\frac{{{\rm{C}}}_{0}-{{\rm{C}}}_{{\rm{t}}}}{{{\rm{C}}}_{0}}\times 100$$

2 $${\rm{TOC}}\,{\rm{removal}}=\frac{{{\rm{TOC}}}_{0}-{{\rm{TOC}}}_{{\rm{t}}}}{{{\rm{TOC}}}_{0}}\times 100\,$$

where C_0_ represents initial concentration of aniline, and C_t_ represents the concentration of aniline at time t. TOC_0_ represents the initial TOC value and TOC_t_ represents the value of TOC solution at time t.

The pH_ZPC_ of the as-fabricated sample was calculated by adding 0.1 g of catalyst sample to 25 mL of NaCl solution. Then, the pH of the solution was adjusted by adding specific amounts of HCl or NaOH to reach to the pH in the range of 2–12. After that, the solutions with different pH values were shaken constantly for 48 hour. Finally, the pH_ZPC_ of the catalyst was obtained similar to the initial pH value.

## Results and discussion

### Characterization of nanocomposites

**Figure 1:**
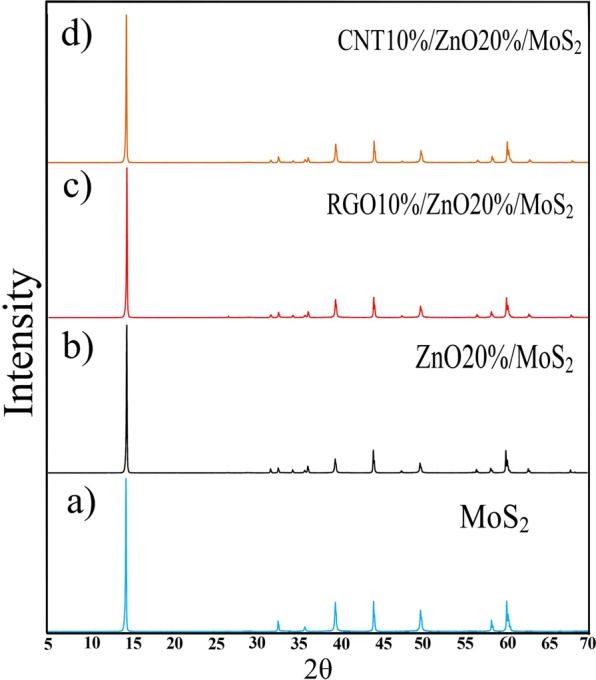
XRD patterns for (**a**) MoS_2_, (**b**) ZnO20%/MoS_2_, (**c**) RGO10%/ZnO/MoS_2_, and (**d**) CNT10%/ZnO/MoS_2_.

In order to characterize the crystallinity and phase purity of MoS_2_, ZnO20%/MoS_2_, RGO10%/ZnO/MoS_2_ and CNTs10%/ZnO/MoS_2_ nanocomposite samples, their XRD patterns were evaluated. Figure 1 shows the XRD patterns related to the as-fabricated samples. Figure 1(a), shows that bare MoS_2_ clearly showed the diffraction peaks at 2*θ* = 14.49°, 32.68°, 35.9°, 39.56°, 44.1°, 49.84°, 58.32°, and 60.14° which could be attributed to (002), (100), (102), (103), (006), (105), (110) and (112), respectively, and they represented the hexagonal structure of MoS_2_ (JCPDS No. 37–1492)^32^. The abovementioned characteristic peaks of MoS_2_ were also observed in the XRD pattern for binary and ternary nanocomposites (Fig. 1(b–d)). Figure 1(b–d) shows that multiple peaks of ZnO were also observed in these nanocomposite samples at 2*θ* = 31.82°, 34.5°, 36.3°, 47.62°, 56.68°, 63.08°, and 68° which are assigned to (100), (002), (101), (102), (110), (103), and (112) lattice planes of hexagonal Wurtzite structure of ZnO (JCPDS No. 36–1451)^37^. In the XRD pattern for RGO/ZnO/MoS_2_ nanocomposite, the sharp diffraction peak of GO at 2*θ* = 10° did not exist, however; a very weak diffraction peak emerged at 2*θ* = 26° which is attributed to (002) reflections of RGO. This implies that GO sheets were transformed into RGO after hydrothermal reduction (Fig. 1(c)). Figure 1(d) shows that there is not any obvious peak related to CNT. This could be due to the low content and amorphous property of CNT. A broad noticeable diffraction peak was observed at 2*θ* = 14.49° with interlayer space of about 0.61 nm indicating that the samples might compose of a few layers of MoS_2_^55^. In addition, the crystallite sizes of the samples were measured by the Scherrer’s equation. The mean crystallite size of the MoS_2_, ZnO20%/MoS_2_, CNT10%/ZnO/MoS_2_, and RGO10%/ZnO/MoS_2_ samples were found to be equal to 66 nm, 64 nm, 54 nm, and 53 nm, respectively at the highest peak for (002) plane. It can be concluded that, carbonaceous materials caused prevention in the growth of the crystallinity for the MoS_2_ nanosheets.

**Figure 2:**
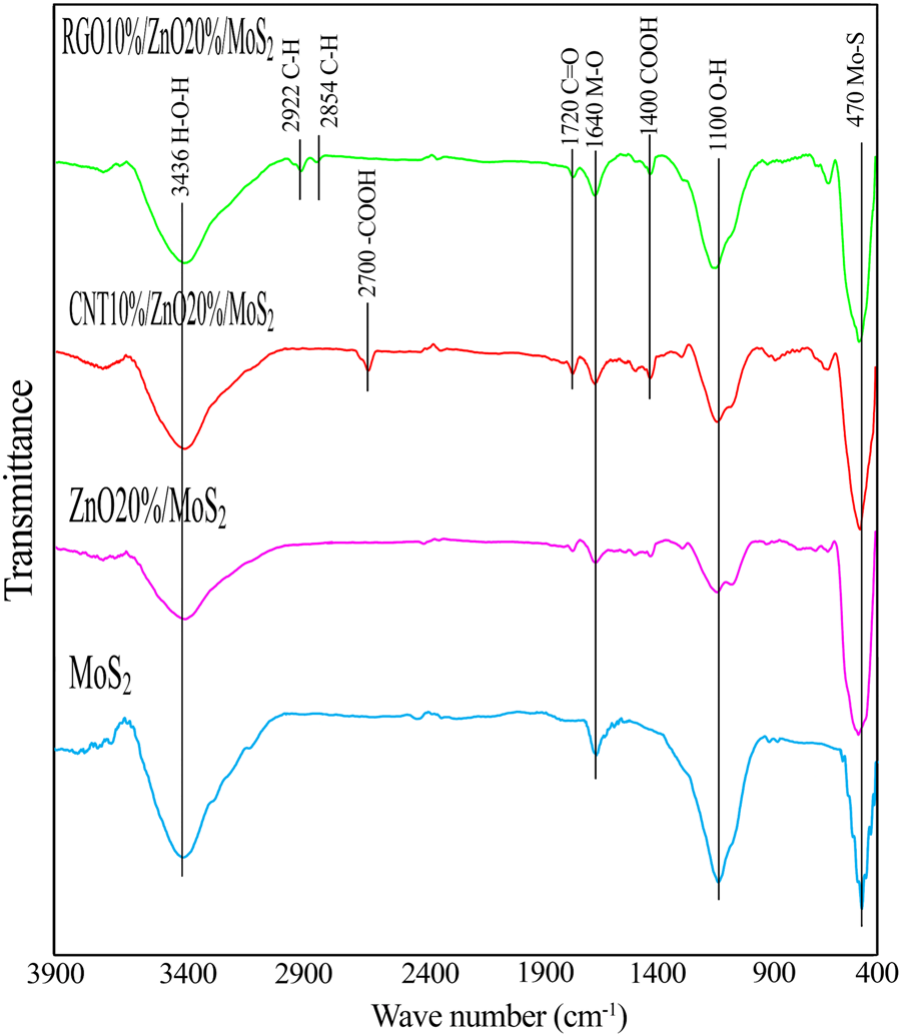
FTIR spectra for (**a**) MoS_2_, (**b**) ZnO20%/MoS_2_, (**c**) CNT10%/ZnO20%/MoS_2_, and (**d**) RGO10%/ZnO20%/MoS_2_.

FTIR was conducted to determine the functional groups in the samples (Fig. 2). The results revealed that MoS_2_ showed the following absorption peaks at 470 cm^−1^, 1100 cm^−1^, 1640 cm^−1^, and 3436 cm^−1^. The absorption peaks at 470 cm^−1^ and 1640 cm^−1^ are assigned to the presence of Mo-S and Mo-O bands in the sample, respectively. The peak at 1100 cm^−1^ and 3436 cm^−1^ were formed as a result of the hydroxyl stretching vibration due to the absorbed water molecules. All these characteristic peaks also existed in the binary and ternary samples. Moreover, in the case of ZnO20%/MoS_2_, the peak of Mo-S bond at 470 cm^−1^ had overlap with the Zn-O peak (at 500 cm^−1^) resulted in formation of a large peak at 470 cm^−1^. In the spectra of CNT10%/ZnO20%/MoS_2_, the peaks at 2700 cm^−1^ and 1720 cm^−1^ were formed due to the vibration of –COOH groups and C=O stretching, respectively. Also, in the spectra of RGO10%/ZnO20%/MoS_2_, the peaks appearing at 2854 cm^−1^ and 2922 cm^−1^ were formed due to the C-H stretching vibration, and the peak centered at 1726 cm^−1^ is corresponded to the C=O stretching frequency from the residual carboxyl groups of RGO^34,37,57–59^.

**Figure 3:**
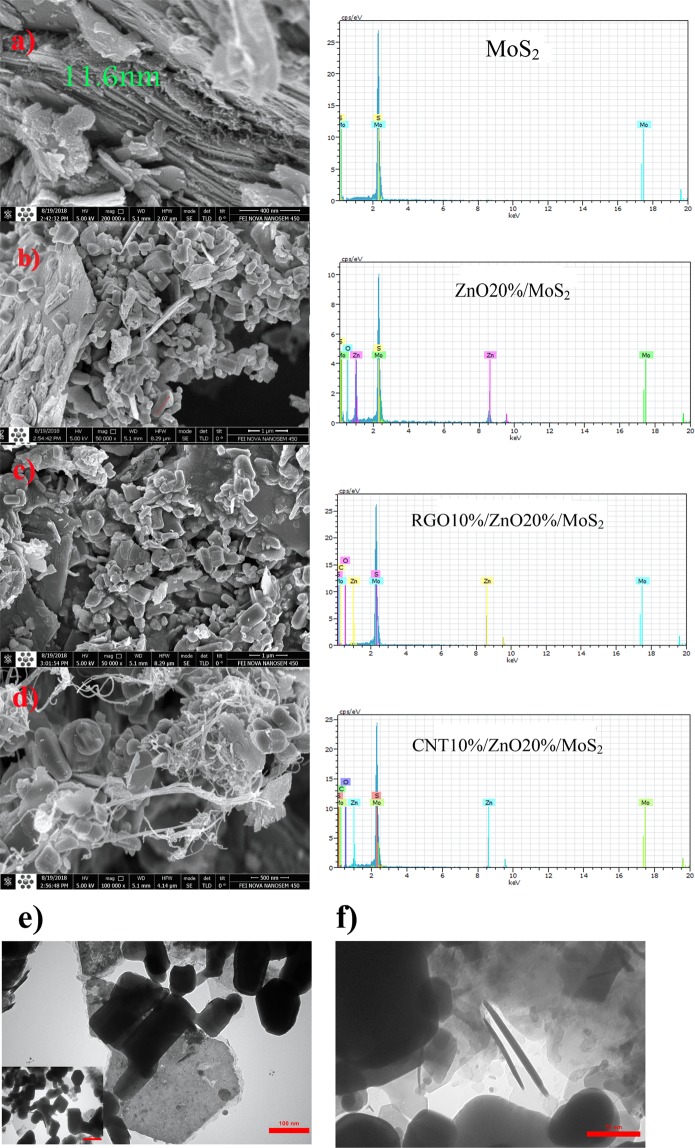
FESEM images along with corresponding EDX results of (**a**) MoS_2_, (**b**) ZnO20%/MoS_2_, (**c**) RGO10%/ZnO20%/MoS_2_, (**d**) CNT10%/ZnO20%/MoS_2_, and TEM images of (**e**) ZnO20%/MoS_2_, (**f**) CNT10%/ZnO20%/MoS_2_.

**Figure 4:**
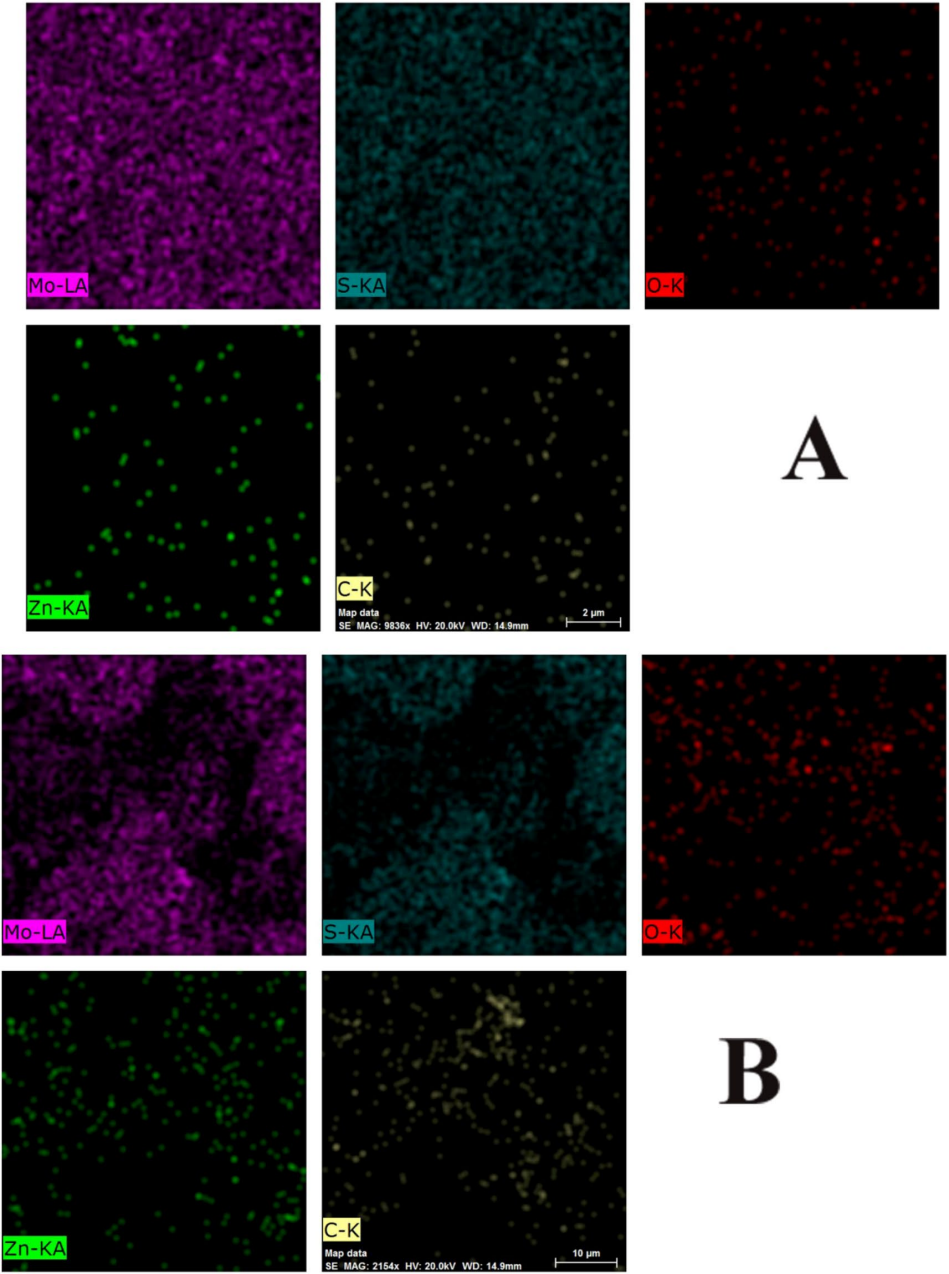
Elemental mapping related to (**A**) RGO10%/ZnO20%/MoS_2_ and (**B**) CNT10%/ZnO20%/MoS_2_.

The FESEM images of the as-fabricated samples along with their corresponding EDX results are presented in Fig. 3(a–d). Figure 3(a) clearly shows the formation of large MoS_2_ nanosheets. As mentioned before, the strong peak at 2*θ* = 14.4 suggests that the fabricated MoS_2_ is few-layered according to the FESEM image of MoS_2._ The average thickness of each sheet is about 11 nm. Figure 3(b) displays the FESEM image of ZnO20%/MoS_2_ nanocomposite. ZnO particles mainly with a rod-like form and hexagonal shape were compactly deposited on the surface of MoS_2_ nanosheets while random dispersion was also observed. For better evaluation of the size and morphology of ZnO particles, at the Fig. 3(e,f) the TEM image is provided which confirmed the FESEM result and indicate that rod-like ZnO nanostructures are distributed on the surface of MoS_2_ nanosheets. It can be seen that the length and diameter of ZnO nanorods are about 100–200 nm and 50–75 nm, respectively. For the sample of CNTs10%/ZnO20%/MoS_2_, CNTs with an average diameter of 20 nm obviously adhered to the ZnO20%/MoS_2_ material which is considered to be beneficial for efficient charge transfer. Finally, in the RGO10%/ZnO20%/MoS_2_ structure, after incorporation of RGO nanosheets, the morphology of the MoS_2_ nanosheets and ZnO nanorods was not changed. Instead, they were embedded into the MoS_2_ nanosheets, leading to provision of enlarged surface area and improvement of electrical performance for the ternary sample. The results of EDX analysis only showed the peaks for Mo, S, Zn, O, and carbon elements, which confirmed the successful formation of all nanocomposite samples and high elemental purity of them. Additionally, elemental mapping was used for further identification of elemental content and distribution of CNTs/ZnO/MoS_2_ and RGO/ZnO/MoS_2_ nanocomposite samples (Fig. 4). Homogeneous distribution of Mo, Zn, C, and O was observed in the ternary composites, indicating well-organized structure with desirable interaction.

**Figure 5:**
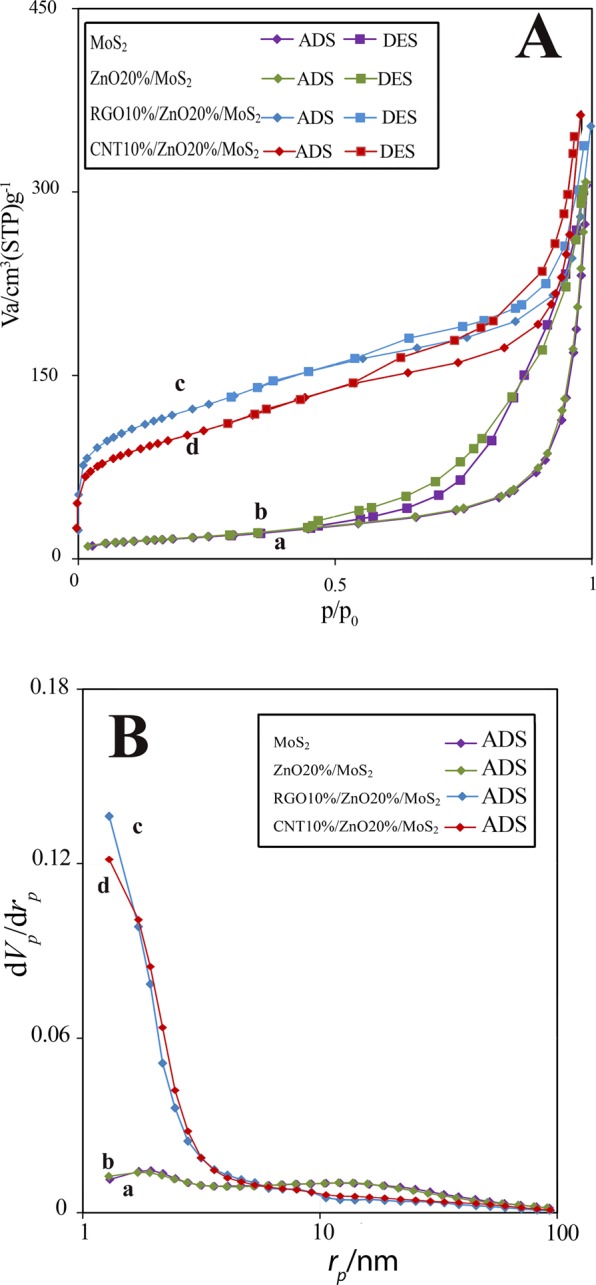
(**A**) N_2_ adsorption-desorption isotherms for (a) MoS_2_, (b) ZnO20%/MoS_2_, (c) RGO10%/ZnO20%/MoS_2_, and (d) CNT10%/ZnO20%/MoS_2_; (**B**) BJH results obtained for (a) MoS_2_, (b) ZnO20%/MoS_2_, (c) RGO10%/ZnO20%/MoS_2_, and (d) CNT10%/ZnO20%/MoS_2_.

The adsorption-desorption isotherm of samples (Fig. 5A), surface area, BJH results (Fig. 5B) and pore sizes (Table 1) were calculated to investigate the effect of ZnO and carbonaceous materials on the enhancement of surface area for the MoS_2_. Figure 5A shows that pure MoS_2_ and ZnO20%/MoS_2_ both showed similar type V isotherms with H_3_ type hysteresis loop, while the ZnO/MoS_2_-carbon materials showed type IV isotherms. These findings indicated that the pore volumes are supplied by mesopores in all samples providing efficient channels for mass transport and enhancing the interface contacts between catalyst surface and pollutant. Pure MoS_2_ had a surface area of 59.94 m^2^g^−1^ and the addition of ZnO nanorods resulted in a slight rise in the surface area, yielding a value of 60.95 m^2^g^−1^ for ZnO20%/MoS_2_. After modification using carbonaceous materials, the BET surface area reached 344.97 m^2^g^−1^ and 420.75 m^2^g^−1^ for CNT10%/ZnO20%/MoS_2_ and RGO10%/ZNO20%/MoS_2_, respectively, indicating that carbonaceous materials especially RGO are considered to be excellent support material for ZnO/MoS_2_. The pore size studies revealed that the average pore size of ternary composites was lower than that of pure MoS_2_ and binary composite. However, the extended surface area of the ternary photocatalysts is still capable to supply more active sites for the adsorption of aniline and enhancing the photocatalytic performance^52^.

**Table 1:** Characteristics of the as-fabricated samples according to the results obtained from BET method.

Catalyst	a_s, BET_	Total pore volume (cm^3^g^−1^)	Mean pore diameter (nm)
MoS_2_	59.94	0.4698	31.349
ZnO20%/MoS_2_	60.95	0.4722	30.993
CNT10%/ZnO20%/MoS_2_	344.97	0.5106	5.921
RGO10%/ZnO20%/MoS_2_	420.75	0.5028	4.780

**Figure 6:**
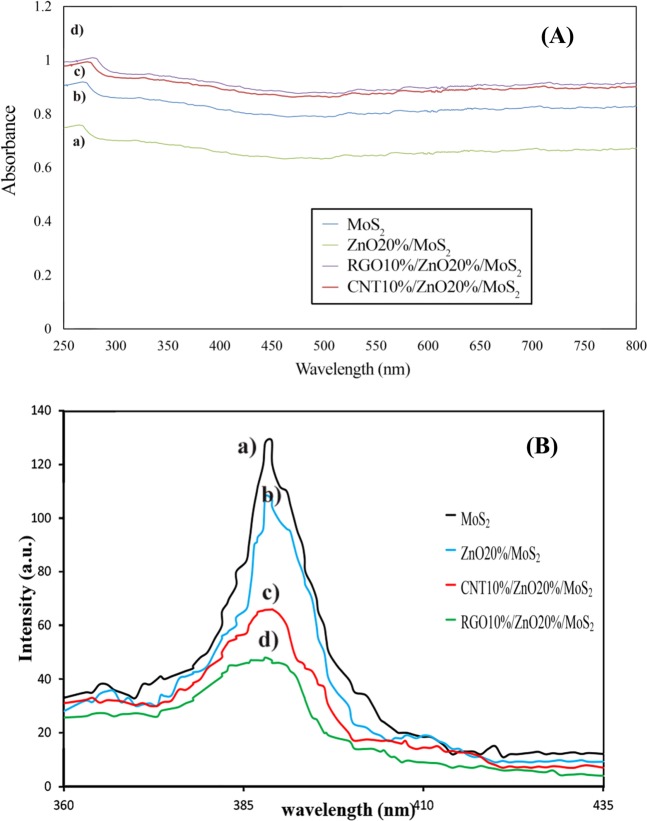
(**A**) UV-visible and **(B)** PL spectra for; (a) MoS_2_, (b) ZnO20%/MoS_2_, (c) CNT10%/ZnO20%/MoS_2_, and (d) RGO10%/ZnO20%/MoS_2_.

The UV-vis analysis of as-fabricated photocatalysts was performed to investigate the optical properties. Figure 6(A) shows the UV-vis adsorption spectra for MoS_2_, ZnO20%/MoS_2_, CNT10%/ZnO20%/MoS_2_ and RGO10%/ZNO20%/MoS_2_ in the range of 190–800 nm. These results are attributed to the color of composite influencing the adsorption capability of the composite powders. Incorporation of ZnO into MoS_2_ catalyst caused a decrease in the cut-off wavelength which has led to higher bandgap value due to color conversion from black to gray for MoS_2_ and ZnO20%/MoS_2_ catalysts, respectively. However, adding graphene and CNT to the final composite caused an increase in the cut-off wavelength. Moreover, the adsorption edge of RGO10%/ZNO20%/MoS_2_ and CNT10%/ZnO20%/MoS_2_ composites showed a red-shift near the wavenumbers of bare MoS_2_, which is attributed to successful interaction between graphene nanosheets and CNT layers. Hence, the presence of RGO or CNT cause the enhancement in the optical response and light adsorption intensity of ZnO20%/MoS_2_ composite which are considered to be influential factors for enhancement of photocatalytic degradation in the industrial plants. The bandgap value for each photocatalyst was calculated using Planck equation:

3 $${{\rm{E}}}_{{\rm{Band}}-{\rm{gap}}}=\frac{{\rm{hc}}}{{\rm{\lambda }}}$$

where, h and c represent the planck constant (4.13566 × 10^−15^) and the light-speed (2.99 × 10^8^ m/s), respectively, and λ represents the cut-off wavelength. The bandgap value and cut-off wavelength for each catalyst is presented in Table 2. The results showed that the bandgap value for photocatalyst containing graphene was higher than that of photocatalyst containing CNT. The obtained results can be attributed to the high charge migration rate between graphene layers and semiconductors and the potential charactristic of graphene in light adsorption^60^.

**Table 2:** The results of bandgap values for the as-fabricated samples.

Catalyst	Cut-off wavelength (λ) (nm)	Bandgap (eV)
MoS_2_	475	2.60
ZnO20%/MoS_2_	500	2.47
CNT10%/ZnO20%/MoS_2_	535	2.31
RGO10%/ZnO20%/MoS_2_	550	2.24

The efficient separation of photo-induced electron-hole pairs is considered as a prominent factor for an ideal photocatalyst. Therefore, the photoluminescence (PL) emission spectra formed as a result of the recombination of photo-excited charge carriers on the surface of the semiconductor was investigated for further study of the migration, separation, and recombination of carriers. The PL spectrum of the bare MoS_2_, binary, and ternary composites are illustrated in Fig. 6(B). Bare MoS_2_ showed an intense PL emission centered at around 388 nm with an excitation wavelength of 340 nm. The PL spectra for other samples were similar to that of bare MoS_2_, but the presence of ZnO, CNT, and RGO caused to the reduction in their PL intensity. This reduction was found to be significant when the carbonaceous materials were incorporated. This could happen since, carbonaceous materials possess excellent electro-conductivity and high electron storage capacity, therefore; the migration of photo-excited electrons could be accelerated from the conduction band of MoS_2_ to the surface of these materials, and therefore, the recombination of charges would be prevented. RGO10%/ZnO20%/MoS_2_ had lower recombination rate compared to CNT10%/ZnO20%/MoS_2_ which is accounted to be better for photocatalytic reaction^60,61^.

### Photocatalytic degradation of aniline

#### Study on the role of ZnO substrate

For evaluating the catalytic performance of MoS_2_-based composites synthesized with different catalyst materials, aniline solution was selected as a model of organic pollutant to be degraded under the exposure to visible light.

**Figure 7:**
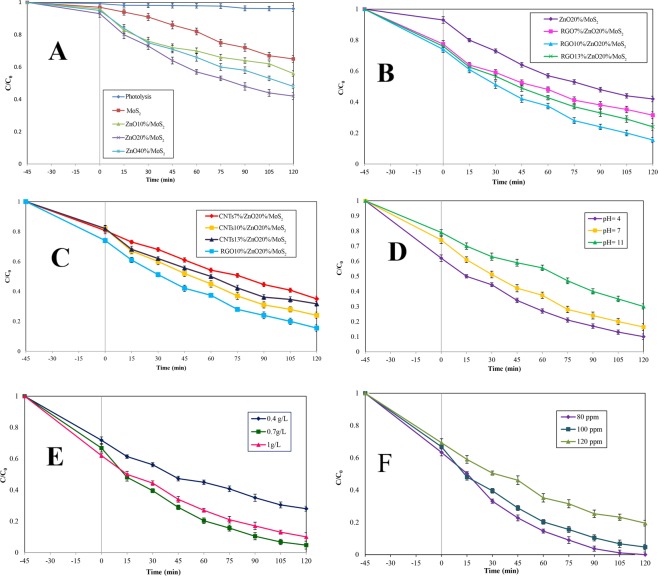
The effects of operational parameters on photocatalytic degradation of aniline after being exposed to visible light irradiation for 120 min, (**A**) Effect of ZnO amount at pH value of 7, catalyst dosage of 1 g/L, aniline concentration of 100 ppm; (**B**) Effect of CNT amount at ZnO content of 20%, pH value of 7, catalyst dosage of 1 g/L, aniline concentration of 100 ppm; (**C**) Effect of RGO amount at ZnO content of 20%, pH value of 7, catalyst dosage of 1 g/L, aniline concentration of 100 ppm; (**D**) Effect of pH value on RGO/ZnO/MoS_2_ at catalyst dosage of 1 g/L, aniline concentration of 100 ppm; (**E**) Effect of RGO10%/ZnO20%/MoS_2_ dosage at pH value of 4, aniline concentration of 100 ppm; and (**F**) Effect of aniline concentration at pH value of 4 and catalyst dosage of 0.7 g/L.

In order to investigate the effect of ZnO substrate, different amounts of ZnO loading (0, 10, 20, and 40 wt.%) were used (Fig. 7A). Furthermore, to investigate the structural stability and photosensitization of aniline under the visible light irradiation, a blank test was carried out in a case without catalyst involvement. Figure 7(A) shows that no obvious self-degradation was observed in the absence of a catalyst, suggesting that aniline is persistent and cannot be degraded under visible light. As expected, in the presence of the catalyst, the single-component had an inadequate degradation rate by 35% which is due to the insufficient absorption sites and fast recombination of charges. The degradation was further improved by loading ZnO content. Increasing the amount of ZnO from 10% to 20% caused an increase in the degradation efficiency from 44% to 58%. However, when the content of ZnO increased to 40%, the catalytic performance decreased to 52%. The reduction in the degradation efficiency of the sample containing the excessive amount of ZnO content might be attributed to the shading effect and increasing opacity of sample blocking the adsorption light of MoS_2_, and high amount of ZnO content could cause severe agglomeration which results in the reduction of charge transfer capability. It can be concluded that, the photo-activity of the samples depends on the mass ratio of MoS_2_ and ZnO, indicating that the proper amount of ZnO is of great importance for the synergistic effect between MoS_2_ and ZnO. Therefore, the selected amount of ZnO content in the ZnO/MoS_2_ is equal to 20 wt.%, and it is considered to be proper for further investigation^62–65^.

#### Study on the role of carbonaceous materials substrate

In order to investigate the effect of carbonaceous materials as support material for catalytic behavior of ZnO20%/MoS_2_, the degradation of aniline was evaluated. Figure 7(B) clearly shows that the presence of RGO had a substantial effect on the photocatalytic ability of ZnO20%/MoS_2_. This result is attributed to the excellent properties of RGO nanosheets incorporated into ZnO20%/MoS_2_ which are explained as follows: (1) incorporation of RGO caused an increase in the surface area and enhancement in the adsorption capacity through providing a 2D support material for ZnO20%/MoS_2_. As a result, more pollutant molecules can be absorbed through absorption sites. (2) More importantly, incorporation of RGO would result in a delay in the recombination of charges by separating them effectively. This delay could be attributed to the characteristics of carbonaceous materials including having remarkable electron storage capacity and the capability to act as the electron sink, such that photo-induced electrons can be accumulated on their structures. Subsequently, these electrons can be trapped by the dissolved oxygen to form superoxide and hydroxide radicals. Concurrently, the holes in the VB could be reacted with the pollutants to degrade them or react with water molecules and produce more.OH radical. With the increase in RGO content from 7% to 10%, the degradation increased gradually from 68.5% to 84%, while by increasing RGO substrate to 13%, the degradation rate was observed to have a decreasing trend (Fig. 7B).

The major reasons for a decreased catalytic ability caused by excessive incorporation of RGO included the role of RGO as a center of recombination of charges and also the shielding effect of the overloaded RGO causing less light to reach to the surface of the catalyst. Same performance was observed after modification of ZnO20%/MoS_2_ using CNTs (Fig. 7C). The comparison made between photo-activity performances of ternary nanocomposites showed that RGO is a better alternative for promoting the efficiency of ZnO20%/MoS_2_ nanocomposite. Although graphene and CNT have similar superior properties in common, such as great adsorption capacity, high electron transition rate, and large specific surface area, they were not similar in enhancing the photocatalytic performance, when used as a composite with ZnO/MoS_2_. After being exposed to 120 min of irradiation, degradation rate of aniline over the optimized RGO10%/ZnO20%/MoS_2_ reached to 84% while for CNT10%/ZnO20%/MoS_2_, this value was equal to 76%. Adsorption capacity of the composite sample is one of the major factors which influence its behavior. Obviously, the adsorption capacity of RGO modified ZnO/MoS_2_ is higher than that of CNTs modified ZnO/MoS_2_ catalyst, which should be due to its larger S_BET_. Based on the optical studies, slight narrowed band gap of RGO10%/ZnO20%/MoS_2_ compared with CNT10%/ZnO20%/MoS_2_ suggests that interaction between RGO and ZnO/MoS_2_ is stronger than that of CNT modified ZnO/MoS_2_^51,66^.

In addition, the PL studies confirmed the improved light-harvesting properties in RGO10%/ZnO20%/MoS_2_. Charge carrier transfer studies provided more evidence regarding the effective migration and separation of charges as well as a reduction in the recombination of charges in RGO10%/ZnO20%/MoS_2_ compared to CNT10%/ZnO20%/MoS_2_. All these findings strongly suggest that RGO has more effect on the photo-degradation of aniline under visible light than CNTs.

In order to make a quantitative comparison between the photo-activity of all nanocomposite samples, the kinetics study was carried out. The photocatalytic degradation of aniline was found to follow the pseudo-first order equation (Fig. S1(A)).

4 $$\mathrm{ln}\left(\frac{{C}_{0}}{C}\right)=kt$$

where C_0_ represents the initial concentration of aniline, C represents the concentration of aniline at time t, and k represents the apparent reaction rate constant.

The rate constants values was obtained from Fig. S1(B). According to the k value, RGO10%/ZnO20%/MoS_2_ sample showed a rapid increase in photo-degradation rate suggesting that RGO is beneficial for promoting the photo-activity. As a result, this nanocomposite was chosen as the desired ternary sample for the following investigation.

#### Study on the role of pH

Solution pH is considered as one of the main parameters that can dramatically influence the photocatalytic reaction since it influences the formation of active radicals and the surface charge of the adsorbate. Due to the formation of intermediate species which may alter the pH of the solution, the pH was monitored during the process. Figure 7(D) depicts the degradation profile of aniline as a function of time at different pH values. Clearly, the degradation rate reached to its maximum level in acidic medium, and it decreased along with increasing pH value. This can be explained according to the surface charge of photocatalyst and the state of aniline at different pH values. Typically, the zero point of charge (pH_zpc_) for the RGO10%/ZnO20%/MoS_2_ influenced its surface charge at different pH values. For the RGO10%/ZnO20%/MoS_2_, the pH_zpc_ was reported to be equal to 2.8. Thus, at pH value < 2.8, the surface of the composite is positively charged, while at pH value > 2.8, it is negatively charged. Besides, the acid dissociation constant (pKa) of aniline was reported to be equal to 4.6 meaning that aniline at values below than this value is positively charged and at values above than this value, it is negatively charged. As a consequence, at high value of pH, both aniline and catalyst are negatively charged. Thus, the interaction between them becomes repulsive, resulting in a decrease in the degradation rate. Conversely, at pH = 4, the electrostatic attraction forces between the positively charged aniline and negatively charged RGO10%/ZnO20%/MoS_2_ leads to an increase in the degradation rate. Moreover, the recombination of e-h pairs is less likely to occur in the acidic medium which is considered to be favorable for photocatalytic reaction. It should be noted that due to the excess amount of H^+^ ions at the low value of pH, more electrons migrate to the surface of the photocatalyst to react with O_2_ to generate active species (superoxide and hydroxyl radicals). Prior studies reported a similar effect of pH value on photo-degradation of aniline^67–69^.

#### Study regarding the role of nanocomposite dosage

Catalyst dosage is another key parameter influencing the efficiency of the photocatalytic reaction. To determine the role of catalyst dosage, different amounts of RGO10%/ZnO20%/MoS_2_ were used varying from 0.4 to 1 g/L. The results are shown in Fig. 7(E). It was found that by increasing the amount of catalyst dosage from 0.4 g/L to 0.7 g/L, the degradation rate increased from 72% to 95%. However, beyond this value, a slight decrease was observed in the rate of reaction. The increase in the degradation rate caused by increasing the catalyst dosage was accompanied by the abundance of active sites, and the generation of active radicals. In higher amounts of catalyst dosage, the decrease in the activity was observed, which is due to the fact that the number of aniline molecules are not enough in the reaction medium to be absorbed on the semiconductor sites. In other words, the additional amount of catalyst substrates does not have any effect in the reaction. Moreover, when high amounts of catalyst is used, the reaction mixture becomes opaque, and the penetration of light to the surface of substrates would be hindered^70,71^.

#### Study on the role of aniline concentration

After adjusting the catalyst dosage and pH of the solution, in order to determine the proper amount of aniline concentration, the photo-degradation process was carried out using three different concentration of aniline and the results are presented in Fig. 7(F). It was observed that the increase the aniline concentration resulted in the decrease in the removal rate. The trends for degradation of aniline were similar to the trends for degradation of other organic pollutants. A possible explanation for this observation is that, as the concentration of aniline increases, a significant amount of light might be absorbed by aniline molecules rather than by catalyst, which in turn less active species would be produced. Besides, the catalyst sample has a restricted number of active sites which are saturated with aniline molecules. Therefore, the adsorption of OH^−^ on the catalyst surface would be decreased which leads to the reduction in the formation of .OH and .O_2_ radicals. Furthermore, the formation of intermediates during the photocatalytic process influences the overall aniline degradation rate. Since by increasing the initial concentration, the amount of formation of intermediates increases which competitively attach to the surface of the catalyst and also competitively react with oxidant species^70,72^.

Herein, for a better evaluation, the catalytic activity of the as-fabricated nanocomposite in the degradation of aniline was compared with those reported in previous studies (see Table 3). The RGO/ZnO/MoS_2_ showed performance than did the nanomaterials used in previous works, since it decompose higher amount of aniline in less time under visible light. To sum up, RGO/ZnO/MoS_2_ is an efficient photocatalyst for decontamination of aqueous medium containing organic pollutants.

**Table 3:** Comparison of photocatalytic aniline degradation results of this study and open literatures.

Catalyst	Aniline concentration (mg.L^−1^)	Catalyst amount (g.L^−1^)	Degradation	Illumination time (min)	Light source	Ref.
Cr-ZnO	150	—	93%	360	Solar light	^70^
ZnO/ H_3_PMo_12_O_40_	50	0.5	43.8%	180	UV	^76^
TiO_2_	50	0.06	62%	120	UV	^77^
CuO	50	0.05	78%	60	UV	^78^
WO_3_/Cu (II)	150	—	85.44%	180	Visible light	^79^
BiO_1.1_Br_0.8_	50	1	90%	240	Visible light	^80^
Barbituric acid doped carbon nitride (CN-BA)	16	0.66	91.53%	120	Sunlight	^74^
RGO/ZnO/MoS_2_	80	0.7	100%	120	Visible light	This study

#### Mineralization

The TOC analysis was performed to identify the remained intermediates in the reaction medium^73^. Therefore, an experiment was carried out in the optimal aniline degradation condition which is as follows: pH value of 4, RGO10%/ZnO20%/MoS_2_ dosage of 0.7 g/L, and initial aniline concentration of 80 ppm. The complete degradation of aniline was achieved over the time period of 120 min, however, the TOC removal was found to be by 40%, and the complete removal of TOC was obtained during the time period of 210 min because aniline molecules were degraded into other organic intermediates. Figure S2 shows the results obtained during mineralization process of aniline.

#### Study on the effect of radical scavenger

**Figure 8:**
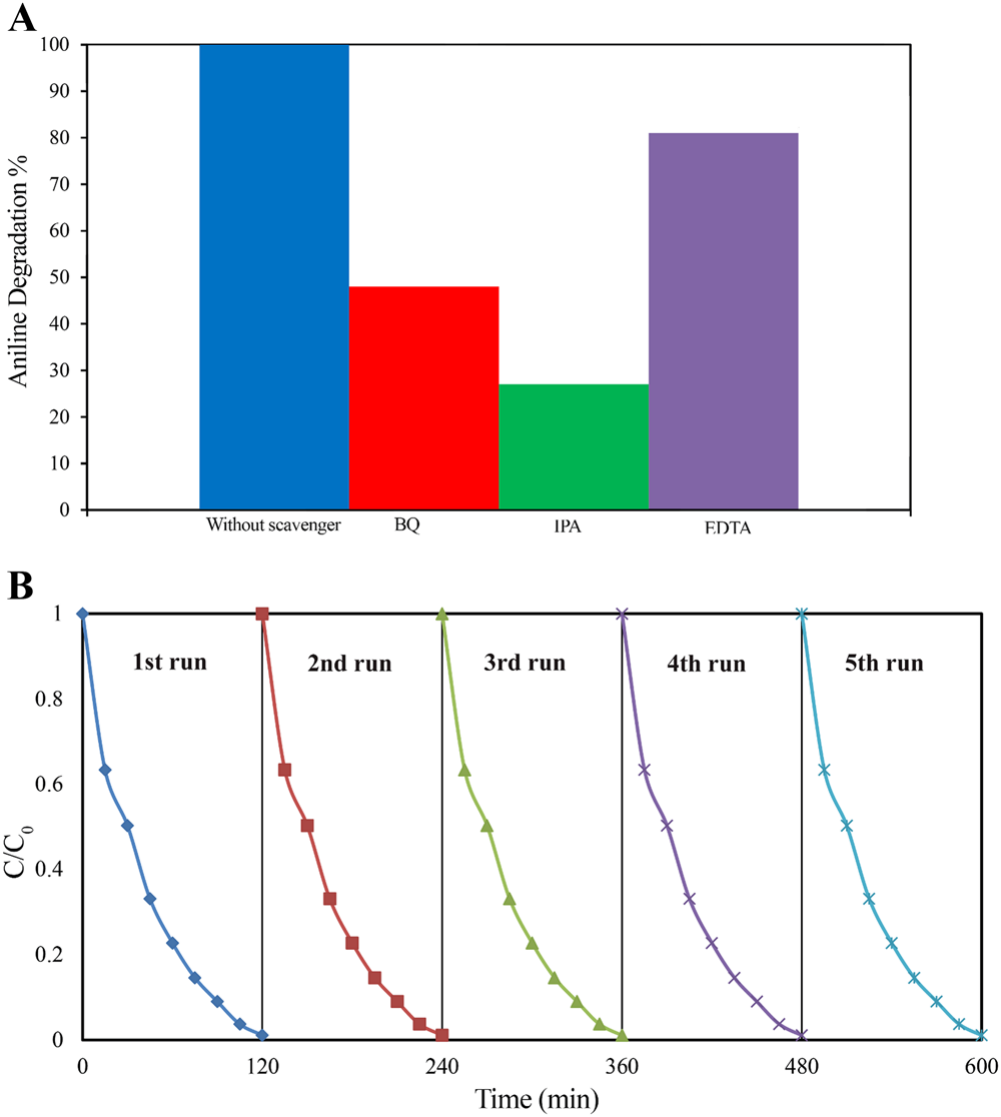
(**A**) Effect of radical scavengers (BQ, IPA, and EDTA) on aniline degradation rate, and (**B**) reusability of RGO/ZnO/MoS_2_ nanocomposite in optimal condition for degradation process of aniline during the five cycles.

Trapping experiments were carried out with the purpose of identifying the active species in degradation of aniline and determining the mechanism of the photocatalytic reaction. Holes (h^+^) and radicals such as hydroxyl radicals and superoxide radicals are the common oxidative species in the photocatalytic process. To identify the predominant species for aniline degradation over the RGO10%/ZnO20%/MoS_2_ nanocomposite in the reaction, three active species of scavengers, namely, disodium ethylene diamine tetra acetic acid (Na-EDTA, 10 mL), benzoquinone (BQ, 10 mL), and 10 mL of isopropanol (IPA, 10 mL) were added to the reaction media for evaluating the effect of radical scavengers h^+^, ^.^O_2_, and ^.^OH, respectively. Figure 8(a) shows that after addition of radical scavengers, the degradation percentage achieved in the order of EDTA > BQ > IPA. EDTA had less effect on the degradation percentage which suggests that h^+^ contributed to the minor extent in the removal of aniline and only a few number of h^+^ species were produced in the degradation process. On the other hand, the reaction efficiency was greatly impeded following the addition of IPA and BQ, suggesting that ^.^OH and ^.^O_2_ are the main active species in the photocatalytic reaction^74^.

According to the abovementioned information, a probable mechanism for degradation of aniline over the RGO/ZnO/MoS_2_ photocatalyst was proposed using the information presented in Fig. S3. Due to the narrow bandgap of MoS_2_, firstly, the electrons are excited from VB of MoS_2_ to the CB. Similarly, ZnO is excited under visible light after modification. Then, the excited electrons in the CB of MoS_2_ migrate to the CB of ZnO. Subsequently, the generated holes in the VB of ZnO migrate to the VB of MoS_2_. The positively charged holes react with water and then translate into hydroxyl radicals. In this case, the charges are successfully separated to avoid being recombined again. Besides, given that RGO has superior electron mobility and storage capacity, it can act as an electron reservoir. It can advance the suppression of electron, hole recombination and extend the lifetime of charge carriers, as confirmed by the PL spectra discussed earlier. The accumulated electrons on the conduction band of ZnO can move to RGO sheets. These electrons can reduce the oxygen into superoxide radicals. These radicals can directly degrade aniline or can be translated to hydroxyl radicals for further degradation of aniline. In conclusion, the synergistic effects of MoS_2_, ZnO, and RGO facilitates the separation of charges and consequently, the degradation of aniline is improved by the use of RGO10%/ZnO20%/MoS_2_.

#### Reusability and stability

In addition to catalytic efficiency, the reproducibility and stability is another essential feature of the photocatalyst, which is referred to its cost-effective practical usage. For investigation of the reusability of the RGO10%/ZnO20%/MoS_2_ sample, the experiment of photocatalytic degradation of aniline at optimal condition (pH value of 4, photocatalytic dosage of 0.7 g/L and aniline concentration of 80 ppm) was repeated for five times. After each run, RGO10%/ZnO20%/MoS_2_ sample was collected using centrifuge, and was washed using distilled water. Then, it was dried, and reused for the next run. Figure 8(B) shows that there is no significant loss of catalytic activity after five cycles of experiment and the negligible loss is maybe due to the loss in the amount of RGO10%/ZnO20%/MoS_2_ during its preparation for each cycle, or reduction of surface area caused by absorbing the untreated by-products to the surface of the photocatalyst. Furthermore, after the five cycles, the sample was subjected to XRD, FTIR, BET, and FESEM analysis to investigate the structural, textural, and morphological stability of RGO10%/ZnO20%/MoS_2_ (Fig. S4(A–D)). The XRD and FTIR patterns of the recovered sample (Fig. S4(A,B)) shows that, no additional peak was appeared and the crystalline nature of photocatalyst has been preserved after five consecutive cycles. Figure S4(C) shows the FESEM image of the nanocomposite after five cycles, and it reveals that the morphological features remained unchanged. Figure S4(D) exhibits the texture properties of the samples after five runs, and it indicates that there was not any obvious change in the type of nitrogen adsorption-desorption isotherms. However, BET measurement data shows slight decrease in the S_BET_ which might be attributed to the fact that during the cycles, a number of untreated intermediates attached on the nanocomposite surface and block the pores, resulting in the reduction of surface area of the nanocomposite.

Consequently, RGO10%/ZnO20%/MoS_2_ showed excellent reusability and stability without any photo-corrosion in the photocatalytic reaction, which makes it to be considered for long-run utilization in the tests for the elimination of organic pollutants.

#### Real wastewater treatment

In order to determine whether the catalyst sample fabricated here was proper for practical purposes, a real petrochemical wastewater sample was prepared to be treated using the RGO10%/ZnO20%/MoS_2_. The properties of petrochemical wastewater sample are presented in Table S1. In order to find the untreated wastewater components, GC-MASS analysis was carried out. The main components of untreated wastewater such as benzene (14.486); 1-Methyl-2-nitro-benzene (15.985); 4-Nitrotoluene (15.985); Aniline (17.773); Phenol (22.781); 3-Methyl-benenamineo (23.845); 4-Chlorophenol (30.510); 2-(5H)-Furanone (31.451); o-Terphenyl (32.092); Naphthalene (32.211); Hexane (34.001); 3-Phenyl-thioethane (34.255); 1,4-Dicyclohexane (34.862); 2-Methyl-benzaldehyde (35.611) and 9,10-Anthracenedione (35.355) (Fig. S5).

The efficiency of the as-fabricated photocatalyst in terms of TOC and COD removal for the petrochemical wastewater sample was found to be equal to 93% and 100%, respectively, in the presence of RGO10%/ZnO20%/MoS_2_ photocatalyst under the optimum degradation condition (pH value of 4, catalyst dosage of 0.7 g/L) over the time period of 440 min (Figs. S6 and S7). Photocatalyst is identified as a reliable material for efficient long term mineralization and partial oxidation of organic compounds^75^.

## Conclusions

In summary, ternary RGO/ZnO/MoS_2_ and CNTs/ZnO/MoS_2_ nanocomposites were synthesized with different amount of RGO and CNTS contents by a typical hydrothermal method and their catalytic ability was evaluated in terms of photo-degradation of aniline. The RGO/ZnO/MoS_2_ showed the best efficiency compared to that of CNTs/ZnO/MoS_2_, ZnO/MoS_2_, and MoS_2_. The outcoming of the present study suggested that RGO has a substantial effect on the catalytic behavior of ZnO/MoS_2_. The results of characterization also confirmed the significant effect regarding the incorporation of RGO. The UV-vis analysis and PL spectra showed that RGO/ZnO/MoS_2_ possess extended visible absorption, and as a result the separation of photo-induced charges was promoted. The BET analysis showed that the surface area also increased greatly in the presence of RGO. In addition, the degradation process was conducted under various operational parameters, and complete degradation of aniline was achieved at pH value of 4, catalyst dosage of 0.7 g/L, and aniline concentration of 80 ppm after being exposed to light irradiation for 120 min. Trapping experiments were performed with the purpose of recognizing active radicals in the reaction and the results suggested that ^.^OH played a paramount role in degradation progress. In the real wastewater sample, the COD and TOC ratios decreased to zero and 7%, respectively after 440 min under the same operational conditions. The obtained results revealed that RGO/ZnO/MoS_2_ has great potential for the remediation of wastewater containing different kinds of organic pollutants due to its tremendous catalytic ability, stability and reusability.

## Supplementary information

Supplementary Information.
