# Supplementary material for: Developing the Ternary ZnO Doped MoS2 Nanostructures Grafted on CNT and Reduced Graphene Oxide (RGO) for Photocatalytic Degradation of Aniline
Source: Sci Rep. 2020 Mar 10;10:4414. doi: 10.1038/s41598-020-61367-7 (PMC7064525; doi:10.1038/s41598-020-61367-7)
Supplement: Supplementary file 1 — Supplementary Information. [file 41598_2020_61367_MOESM1_ESM.docx]

**Supporting Information**

**Developing the Ternary ZnO Doped MoS_2_ Nanostructures Grafted on CNT and Reduced Graphene Oxide (RGO) for Photocatalytic Degradation of Aniline**

Parisa Ghasemipour^1^, Moslem Fattahi^1*^, Behnam Rasekh^2^, Fatemeh Yazdian^3^,

1. Chemical Engineering Department, Abadan Faculty of Petroleum Engineering, Petroleum University of Technology, Abadan, Iran

2. Microbiology and Biotechnology Research Group, Research Institute of Petroleum Industry, Tehran,
Iran

3. Department of Life Science Engineering, Faculty of New Science and Technologies, University of
Tehran, Tehran, Iran

Email addresses: [parisaghasemi9015@yahoo.com](mailto:parisaghasemi9015@yahoo.com); [fattahi@put.ac.ir](mailto:fattahi@put.ac.ir) (Corresponding author); [rasekhb@ripi.ir](mailto:rasekhb@ripi.ir); [yazdian@ut.ac.ir](mailto:yazdian@ut.ac.ir)

| 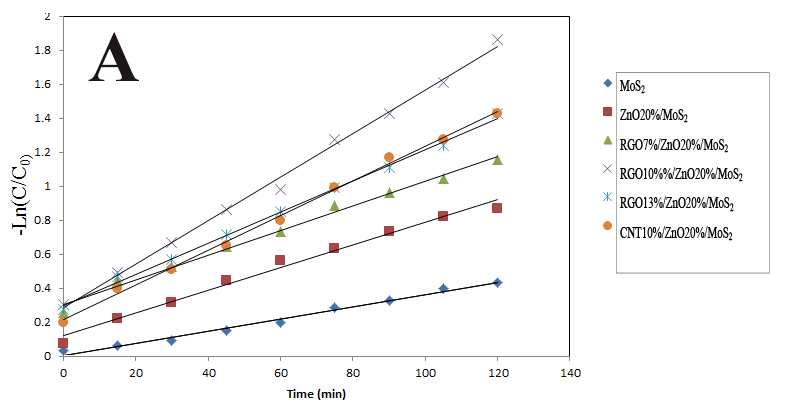 |
| --- |
| **B** |

**Figure S1**: **(A)** kinetics study on degradation of aniline for different samples, **(B)** apparent rate constant for different samples of catalysts


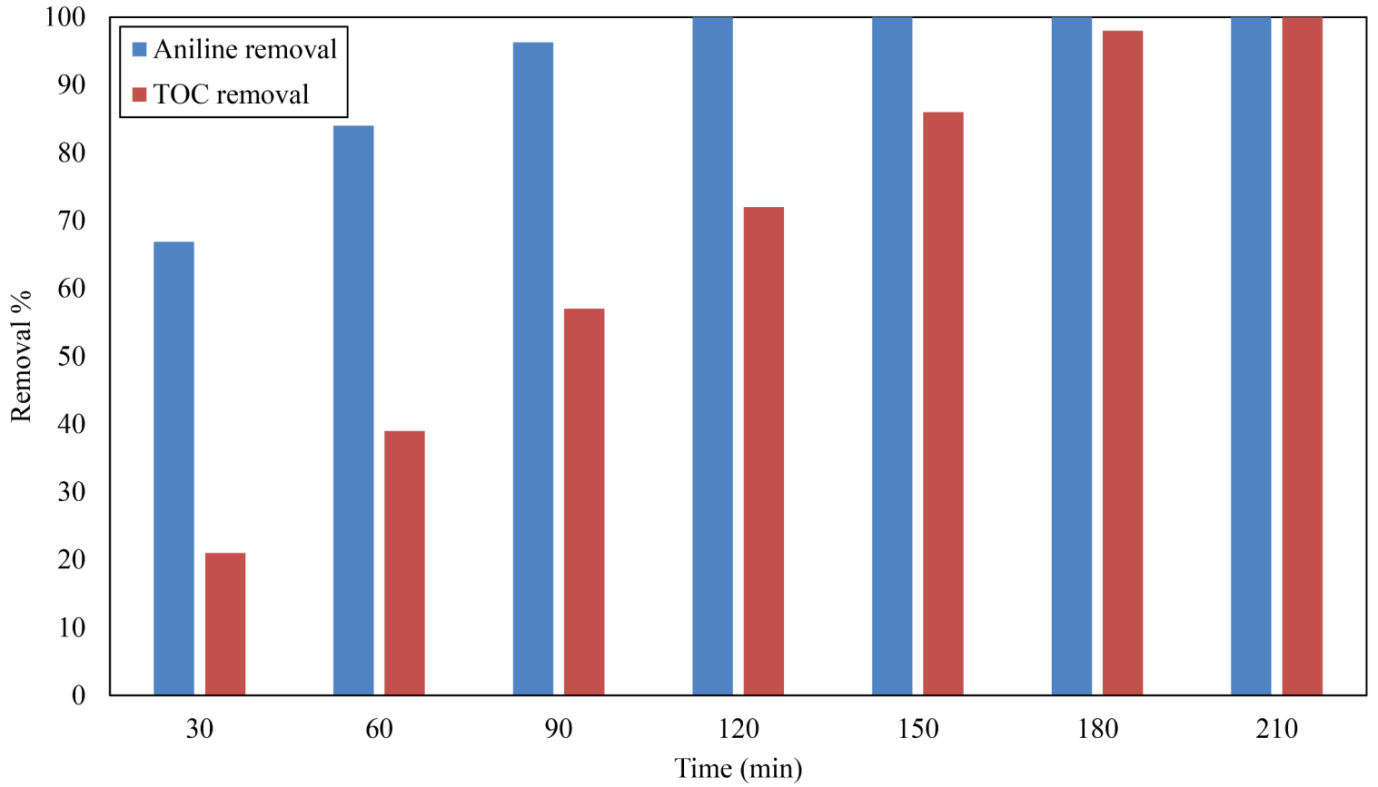


**Figure S2**: Comparison of photocatalytic degradation of aniline and the TOC removal under visible light irradiation


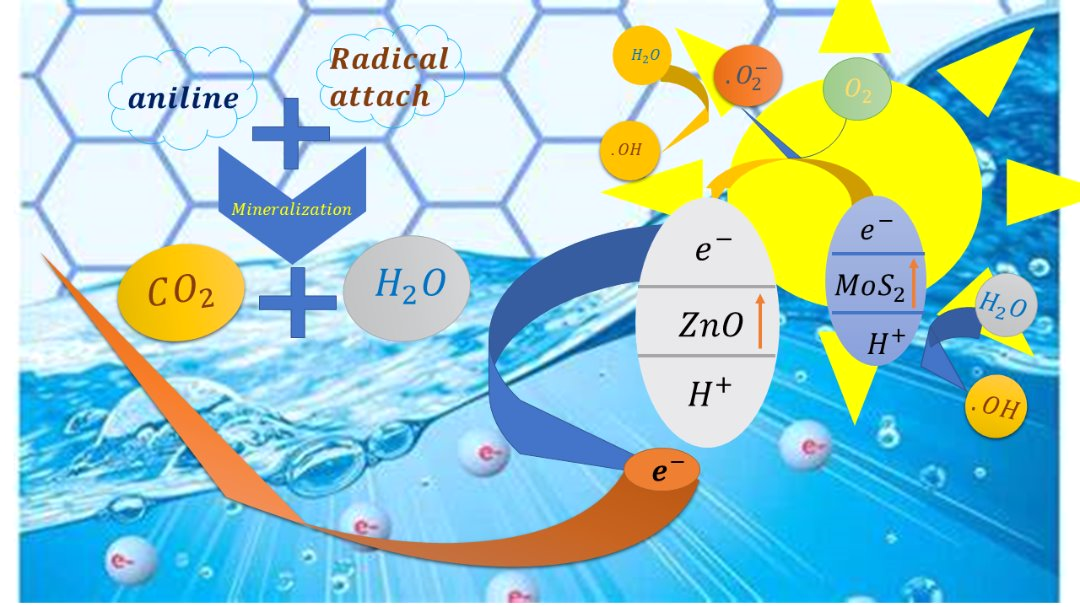


**Figure S3**: Proposed mechanism for describing the photocatalytic degradation of aniline under visible light irradiation on the RGO10%/ZnO20%/MoS_2_.

| 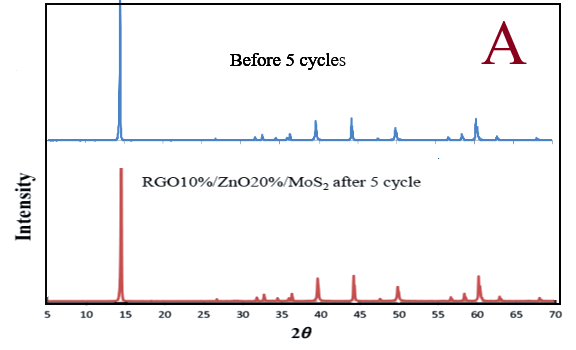 |
| --- |
| 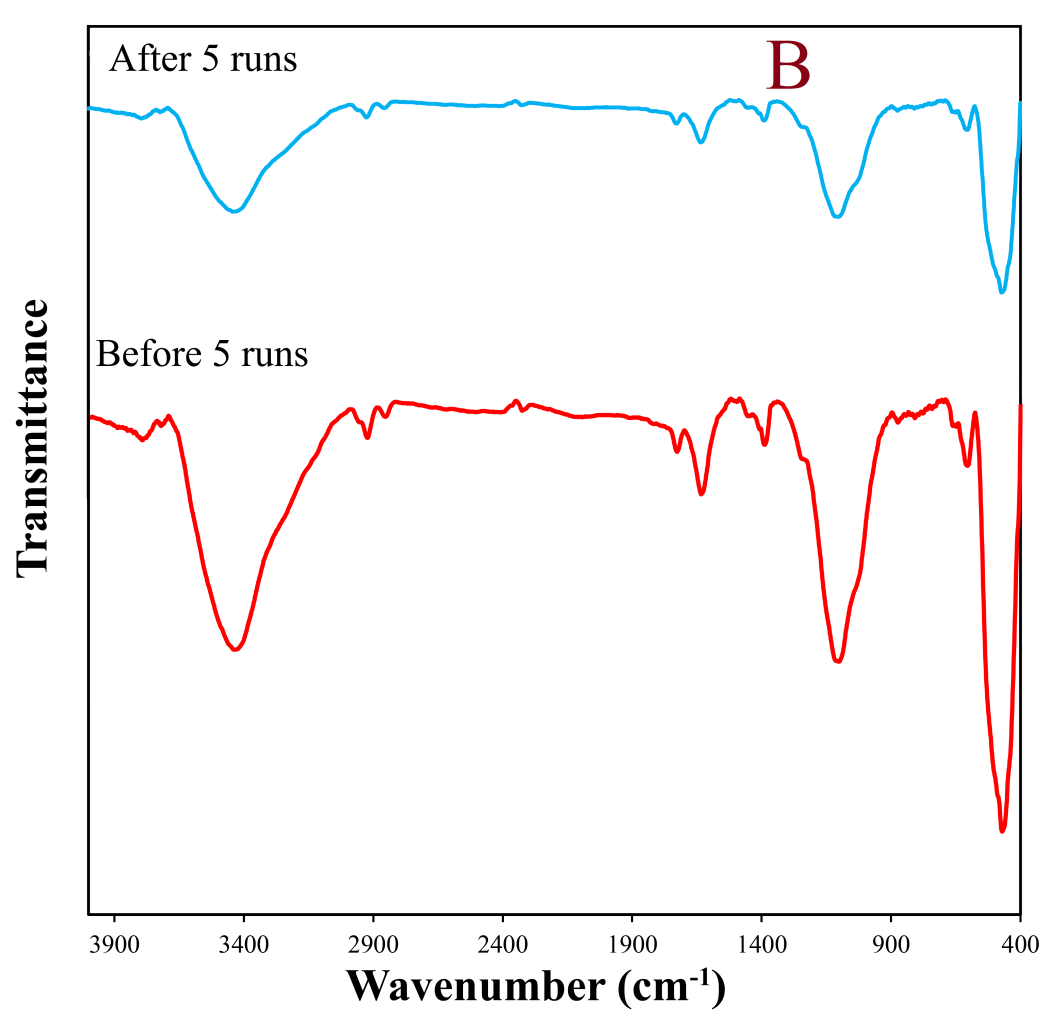 |
| 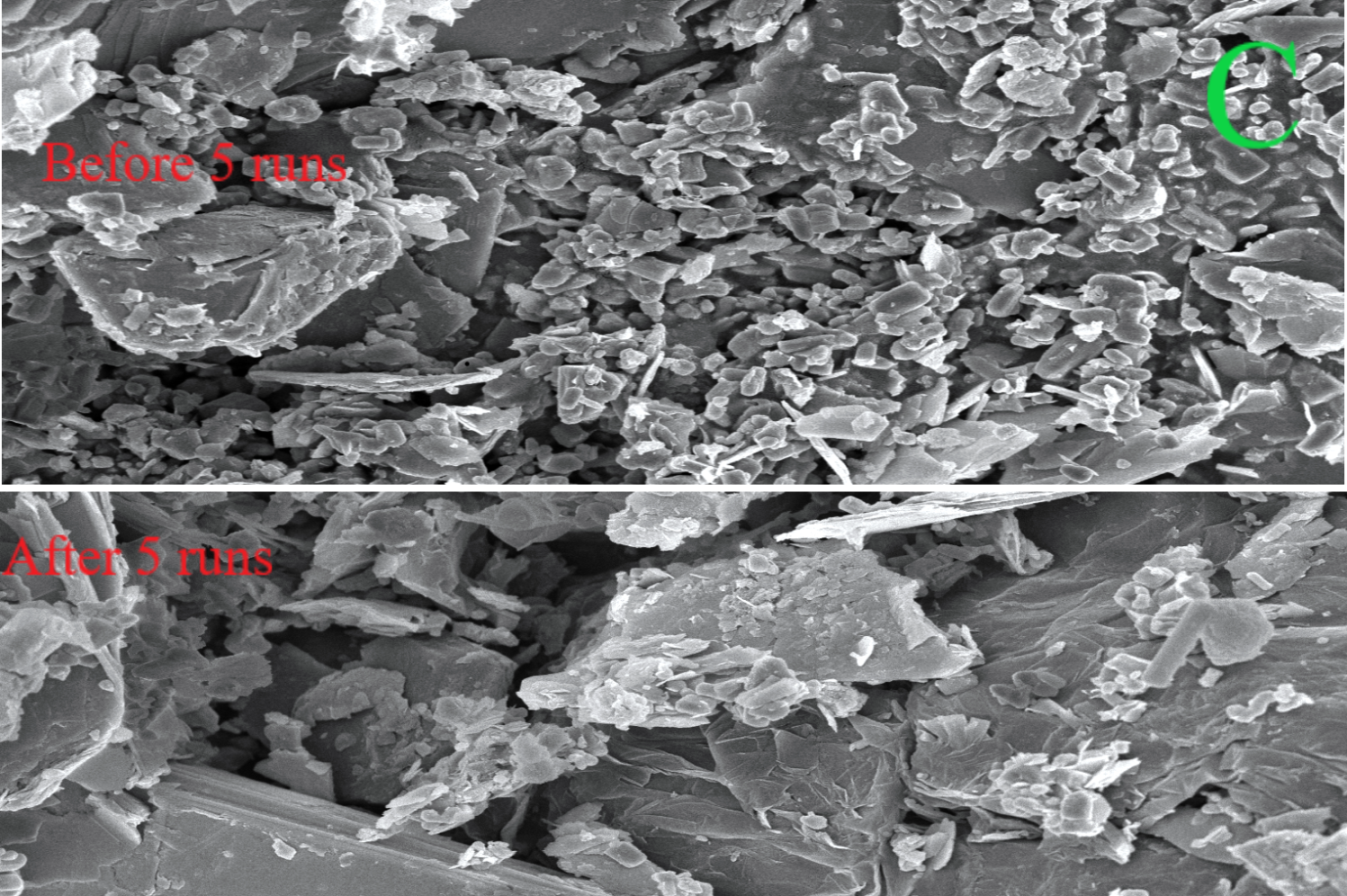 |
| 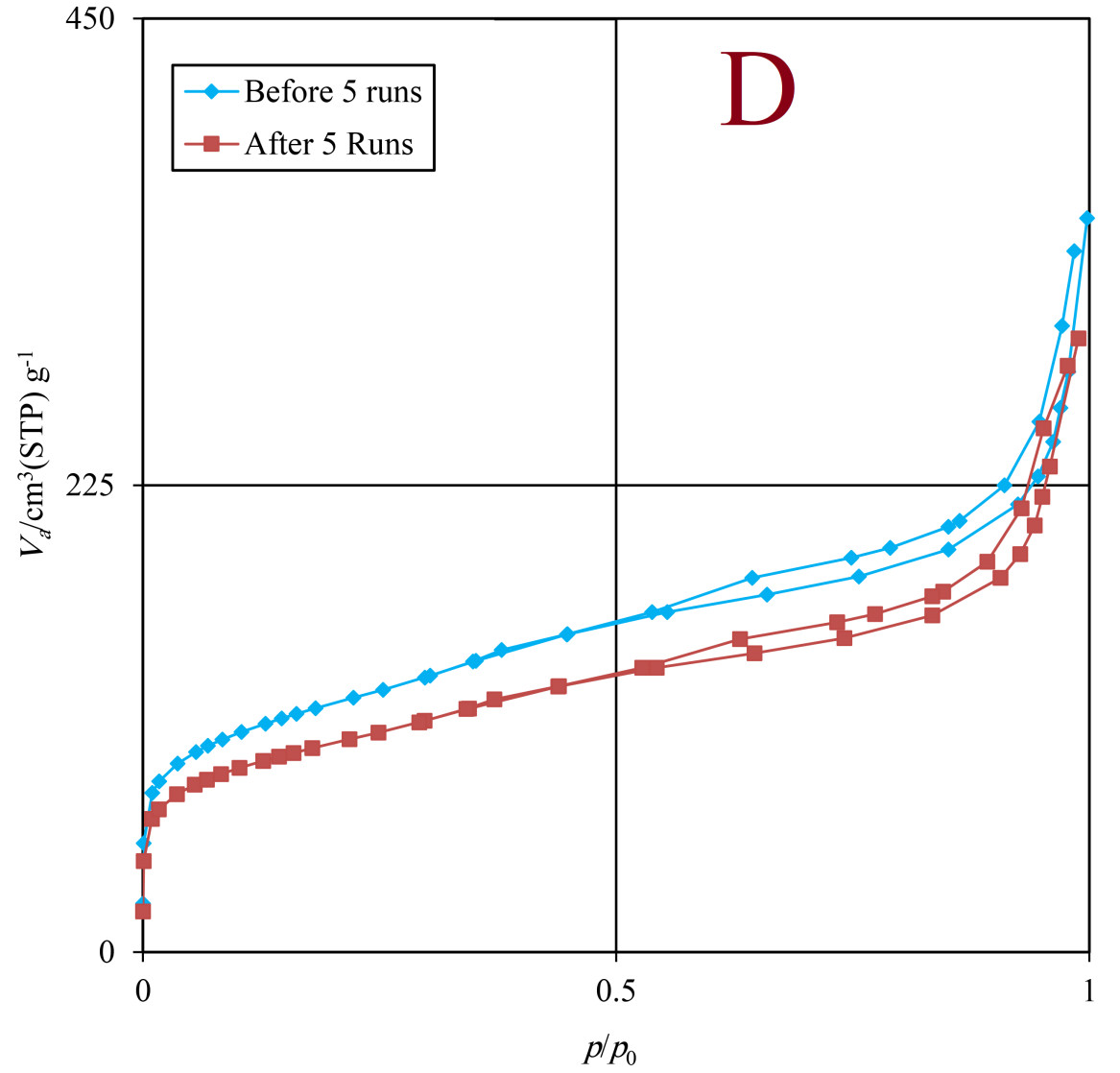 |

**Figure S4**: (A) XRD pattern of RGO/ZnO/MoS_2_ catalyst before and after five cycles, (B) FTIR pattern of RGO/ZnO/MoS_2_ catalyst before and after five cycles, (C) FESEM image of RGO/ZnO/MoS_2_ catalyst before and after five cycles, (D) BET results of RGO/ZnO/MoS_2_ catalyst before and after five cycles.

**Table S1:** Properties of wastewater sample used in this study

| **Parameter** | **Value range** | **Average** |
| --- | --- | --- |
| Total COD (mg/L) | 1335-1884 | 1110 |
| BOD_5_ (mg/L) | 90-132 | 111 |
| BOD_5_/COD | - | 0.082 |
| TOC (mg/L) | 767-1243 | 1005 |
| Turbidity (NTU) | 12-18 | 15 |
| TDS (mg/L) | 1509-1961 | 1580 |
| TSS (mg/L) | 105-178 | 162 |
| pH | 6.8-7.6 | 7.2 |


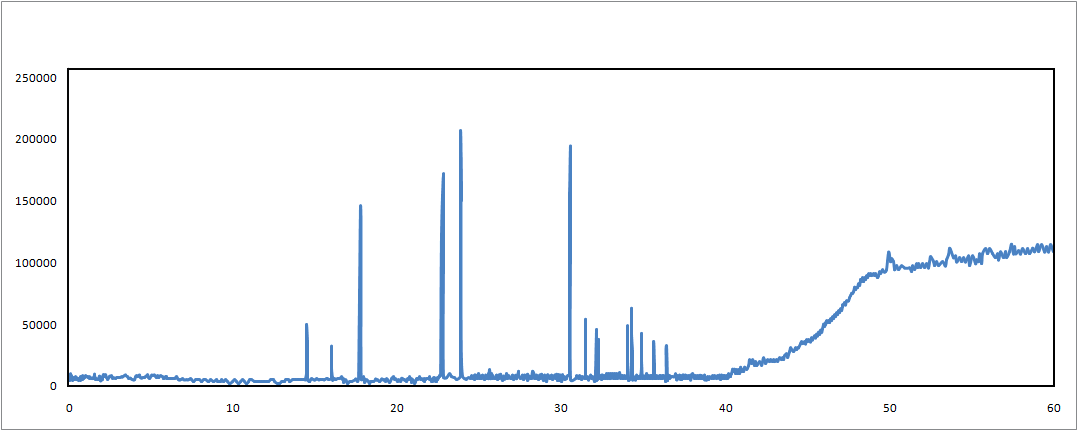


**Figure S5**: GC-MASS analysis of an untreated petrochemical wastewater.

**Figure S6**: TOC removal rate for real wastewater sample.

**Figure S7**: COD removal rate for real wastewater sample.
